# Effects of maternal genotypic identity and genetic diversity of the red mangrove *Rhizophora mangle* on associated soil bacterial communities: A field‐based experiment

**DOI:** 10.1002/ece3.6989

**Published:** 2020-11-10

**Authors:** Hayley Craig, John Paul Kennedy, Donna J. Devlin, Richard D. Bardgett, Jennifer K. Rowntree

**Affiliations:** ^1^ Department of Earth and Environmental Sciences The University of Manchester Manchester UK; ^2^ Department of Natural Sciences, Ecology and Environment Research Centre Manchester Metropolitan University Manchester UK; ^3^ Smithsonian Marine Station Fort Pierce FL USA; ^4^ Department of Life Sciences Texas A&M University Corpus Christi Corpus Christi TX USA

**Keywords:** associated species, intraspecific diversity, plant genotype, plant‐bacterial interactions

## Abstract

Loss of plant biodiversity can result in reduced abundance and diversity of associated species with implications for ecosystem functioning. In ecosystems low in plant species diversity, such as Neotropical mangrove forests, it is thought that genetic diversity within the dominant plant species could play an important role in shaping associated communities. Here, we used a manipulative field experiment to study the effects of maternal genotypic identity and genetic diversity of the red mangrove *Rhizophora mangle* on the composition and richness of associated soil bacterial communities. Using terminal restriction fragment length polymorphism (T‐RFLP) community fingerprinting, we found that bacterial community composition differed among *R. mangle* maternal genotypes but not with genetic diversity. Bacterial taxa richness, total soil nitrogen, and total soil carbon were not significantly affected by maternal genotypic identity or genetic diversity of *R. mangle*. Our findings show that genotype selection in reforestation projects could influence soil bacterial community composition. Further research is needed to determine what impact these bacterial community differences might have on ecosystem processes, such as carbon and nitrogen cycling.

## Graphical abstract

Using a manipulative field experiment, we show that maternal genotype, but not genetic diversity, of red mangrove trees affects associated soil bacterial community composition. This could have important implications for ecosystem processes, such as carbon and nitrogen cycling.

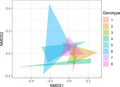

## 1 INTRODUCTION

The world is currently experiencing losses in biodiversity at an accelerated rate (IPBES, 2019), and much research has focussed on understanding the impacts of this loss on ecosystem functioning (Duffy et al., 2017; Hooper et al., 2005; Loreau et al., 2001). As primary producers, plants play a critical role in supporting most terrestrial ecosystems, and there is a general consensus that reductions in plant diversity will lead to decreases in plant productivity (Hooper et al., 2012) with knock on effects for associated species and recycling of essential nutrients (Cardinale et al., 2011; Hooper et al., 2005). While much of the research examining plant diversity–ecosystem functioning relationships has focussed on loss of plant species or functional groups (Balvanera et al., 2006; Hooper et al., 2005; Wardle, 2016), other studies have also found that loss of genetic diversity within a dominant or foundation plant species can have similar consequences for associated species and ecosystem functioning (Crutsinger et al., 2006; Hughes et al., 2008). Genetic diversity can decline more rapidly than species diversity in response to anthropogenic pressures and habitat fragmentation, so it can be considered as a potential indicator for future species losses (Helm et al., 2009).

There can be competitive advantages for genetically diverse populations including enhanced productivity (Crutsinger et al., 2006) and reproductive success (Newman & Pilson, 2006). Genetically diverse populations have also been shown to better utilize available resources, reducing competition between conspecifics (Boyden et al., 2008), and display greater stability (Prieto et al., 2015) and capacity to adapt to environmental change or disturbance (Jump et al., 2009; Reusch et al., 2005). When genetic diversity is low, there are less likely to be individuals with the necessary traits to cope with environmental change, although there are other potential mechanisms to cope with change such as phenotypic plasticity (Gratani, 2014) and epigenetic responses (Verhoeven et al., 2016). Low genetic diversity is also associated with inbreeding, reduced fitness through homozygosity, and increased chance of extinction (Frankham, 2005; Reed & Frankham, 2003).

The level of genetic variation within a plant species can also affect interactions with other species. Crutsinger et al. (2008) found that high intraspecific genetic diversity within populations of the perennial plant *Solidago altissima* can deter biological invasion of other plant species. Thus, loss of genetic diversity can increase susceptibility of a population to invasion. On the other hand, genetic diversity within a parasitic species can increase their successful establishment within species‐rich habitats, as has been observed in populations of the parasitic plant *Rhinanthus minor* (Rowntree & Craig, 2019). Changes in plant genetic diversity can also impact associated species that depend on those plants for food or shelter, with potential cascading effects throughout the food chain. For example, a meta‐analysis of manipulative experiments found that richness and abundance of most arthropod trophic groups generally increase with plant genetic diversity (Koricheva & Hayes, 2018).

To date, most studies of plant genetic diversity effects on associated species have focussed on other plant species or arthropods. There is a dearth of knowledge on how plant genetic diversity might impact associated soil microbial communities. Soil microbial communities provide many important ecosystem functions and services (Bardgett & van de Putten, 2014), and recent studies show that the structure and/or diversity of soil microbial communities significantly relates to soil functionality (Zhou et al., 2020), and processes of nitrogen (Zheng et al., 2019), and carbon cycling (Maron et al., 2018). In a grassland microcosm experiment that manipulated the soil microbiome, microbial richness and network complexity were shown to positively influence several ecosystem functions related to nutrient cycling (Wagg et al., 2019). Reduced microbial diversity is thought to lead to lower levels of soil functioning due to fewer taxa present to support the same function and lower diversity of taxa to support different functions (Wagg et al., 2019). It is therefore important to understand how changes in plant diversity may affect associated soil microbial communities and the functions that they provide. Positive correlations have been observed between interspecific plant diversity and soil microbial richness across a range of biomes (Liu et al., 2020). Few studies have focussed on intraspecific diversity, however, in one study of *Populus* spp stands, relationships were detected between plant genetic diversity and soil microbial community composition, which were associated with changes in exoenzyme activity and nitrogen availability in the soil (Schweitzer et al., 2011).

In addition to genetic diversity, the identity of genotypes or phenotypes within populations of primary producers has been shown to affect ecosystem functions associated with nutrient cycling (Raffard et al., 2019). Many studies have detected differences in soil microbial community composition among plant genotypes (e.g., Aira et al., 2010; Gallart et al., 2018; Schweitzer et al., 2008; Shenton et al., 2016). Purahong et al. (2016) found effects of both genetic diversity and tree genotypic identity on soil enzyme activity in a subtropical forest, but the results were not consistent across all four tree species in the study. Plant genotypic effects on soil microbial communities have generally been attributed to differences in the chemical composition of root exudates (Micallef et al., 2009) and quantity and quality of leaf litter among genotypes (Schweitzer et al., 2005), but they could also be related to factors such as variation in root morphology or belowground carbon allocation.

In studies of natural systems, it can be difficult to separate effects of plant intra and interspecific diversity. Therefore, ecosystems that are poor in plant species could be better suited for studying intraspecific genetic diversity without the confounding effect of species diversity. Neotropical mangroves typically have few plant species, with only three true mangrove tree species found in Florida (Spalding et al., 2010), and the distribution of these vary spatially across the tidal zone (McKee, 1993). In these species‐poor environments, it has been suggested that plant intraspecific genetic diversity may play an important role in shaping the communities of mangrove associate species and ecosystem functioning (Farnsworth, 1998). While we are not aware of any studies that have tested effects of intraspecific genetic diversity in mangrove ecosystems, maternal genotype has been found to influence both resistance to mortality from infestation by the parasitic beetle, *Coccotrypes rhizophorae* (Devlin, 2004), and seedling survival in different positions in the intertidal zone (Proffitt & Travis, 2010) in the red mangrove, *Rhizophora mangle*. These results demonstrate how genotypic identity or reduction in genetic diversity could impact the ability of mangrove plant populations to deal with pressures such as herbivory or sea level rise.

Mangroves are considered to be important coastal ecosystems for carbon storage (Alongi, 2012; Donato et al., 2011), as their waterlogged soils have low rates of decomposition (Cebrian, 1999), resulting in belowground carbon stocks that are several times greater than that of other forest types (Donato et al., 2011). Human activities can contribute to the loss of plant genotypes and genetic diversity in mangroves through disturbance (Salas‐Leiva et al., 2009) and fragmentation of populations (Granado et al., 2018). If loss of plant genetic diversity then affects the composition of soil microbial communities, this could have implications for soil functioning leading to changes in carbon storage rates and emissions of greenhouse gases, including carbon dioxide, nitrous oxide, and methane.

Here, our aim was to investigate how genotypic identity and genetic diversity within the mangrove tree species *R. mangle* affects the composition and richness of its associated soil bacterial community as determined using terminal restriction fragment length polymorphisms (T‐RFLP). We hypothesized that: (a) maternal genotype would be an important determinant of soil bacterial community composition; and (b) *R. mangle* populations of higher genetic diversity would support richer soil bacterial communities. This was tested in a field‐based experiment where plots were planted with three levels of *R. mangle* genotypic diversity using a pool of eight maternal genotypes. We focus on bacterial communities as they dominate the mangrove soil microbiome (Alongi, 1988; Andreote et al., 2012) and are responsible for most of the soil carbon flux in these environments (Holguin et al., 2001). We anticipate that our results will provide new insights into the influence of plant genotype and genetic diversity on belowground microbial communities in mangroves, which could help inform the restoration of degraded mangrove sites.

## 2 METHODS

### 2.1 Study site

**FIGURE 1:**
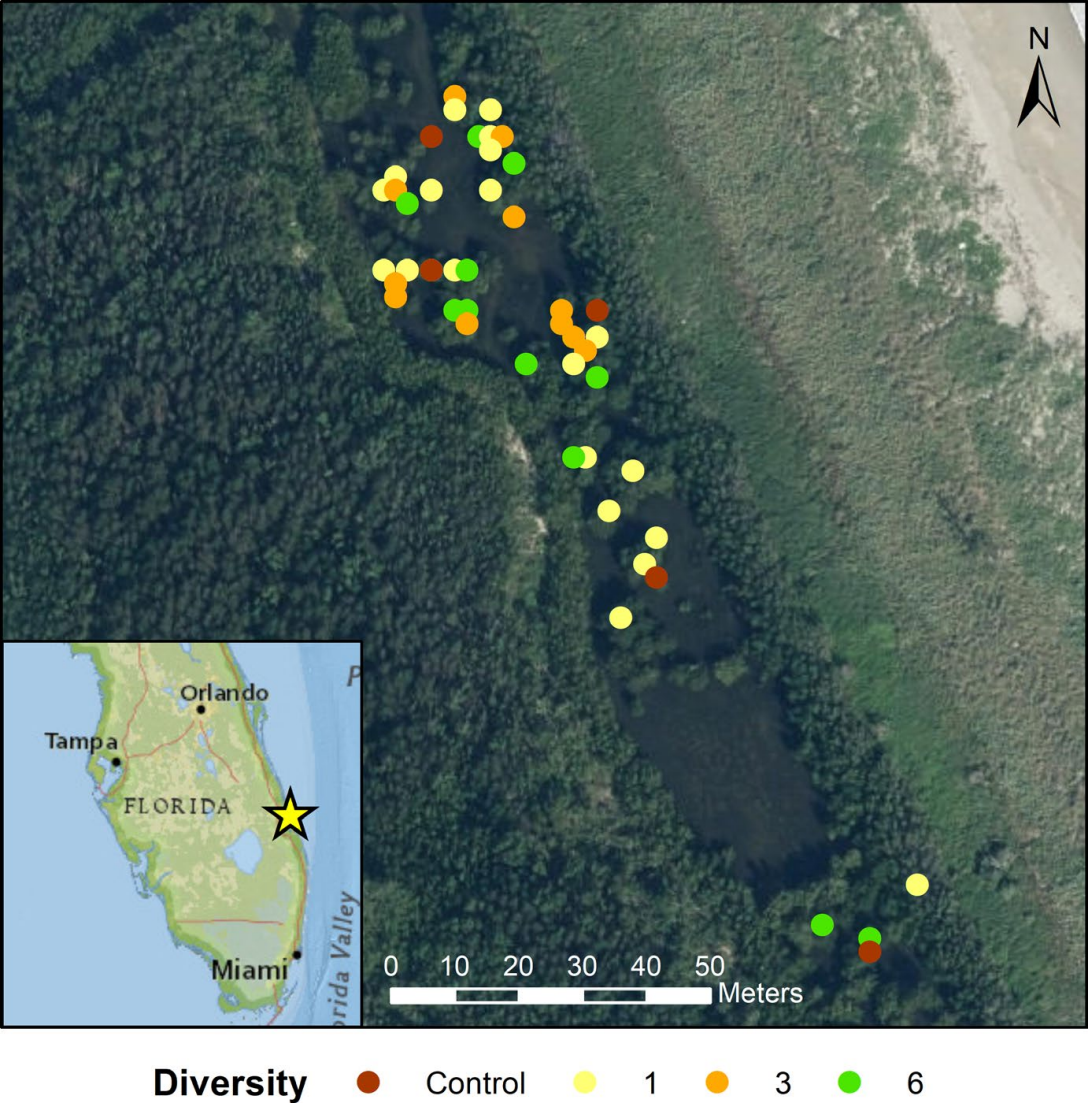
Location of the field experiment site and the experimental plots sampled in this study. Colors of the points show the number of *Rhizophora mangle* genotypes planted within the plots. Plot coordinates ± 5 m accuracy. Map produced in ESRI ArcMap 10.4.1

The study site (Figure 1) is located at the Little Mud Creek mangrove impoundment, on South Hutchinson Island within the Indian River Lagoon system, St. Lucie County, Florida, USA (27.378°N, 80.254°W). The site suffered severe hurricane damage in 2004, when the eyes of the twin hurricanes Frances and Jeanne both hit near the site, pushing the foredune into the impoundment. The impoundment was excavated, and new drainage culverts were installed. At experiment initiation, a few *R. mangle*, *Avicennia germinans* (black mangrove), and *Laguncularia racemosa* (white mangrove), had established naturally at the site, but the mangrove vegetation in the area adjacent to the site is dominated by *R. mangle*. The sediment is sandy with shell fragments, and at the time of sampling, organic matter content in the top 10 cm of sediment in vegetated areas was around 5%–15%. Mean water levels in the Indian River Lagoon are typically higher in late autumn (Smith, 1986) when the site can be inundated for several weeks at a time, but the soil surface is generally exposed at low tide for the rest of the year.

### 2.2 Experimental design

The *R. mangle* genetic diversity field‐based experiment was set up at Little Mud Creek in November 2011. Propagules of *R. mangle*, a viviparous species, were collected from eight maternal trees, four from each of two locations: within Little Mud Creek, close to the experimental site; and adjacent to Harbor Branch Oceanographic Institute (27.534°N, 80.349°W), which is approximately 20 km north northwest of Little Mud Creek. All propagules within a maternal cohort are at least half siblings, but could be more closely related as *R. mangle* commonly self‐fertilizes (Lowenfeld & Klekowski, 1992) and selfing rates of 0.13–0.61 have been observed in *R. mangle* populations near the study area (Kennedy et al., 2017). Maternal cohort was used as a proxy for genotype; hereafter maternal cohorts are referred to as genotypes.

The experiment was set up with three levels of genetic diversity: monocultures of each of the eight genotypes, and varying mixtures of three or six genotypes, selected from a pool of the eight genotypes. The number of genotypes in the experimental pool was based on the number of trees at the collection sites that were mast producing that year (i.e., had sufficient numbers of propagules for suitable replication in the experiment). While no data are available regarding the number of genotypes that may naturally be found at the spatial scale used in this experiment, both reproduction and recruitment of *R. mangle* is generally low in the years following hurricanes (Proffitt et al., 2006). Thus we would not expect genotypic diversity to be as high as has been observed in similar experiments in other systems (e.g., Crutsinger et al., 2008). Experimental plots were randomly assigned to treatments and were established on unvegetated mudflats within the tidal channel. Plot placement was determined on site during planting taking into account water depth and distance from other plots (at least 2 m). Each plot measured 45 x 30 cm and 12 propagules were randomly assigned within a matrix of three rows with 15 cm spacing between each propagule (based on an average distance observed between propagules, which had naturally recruited at a nearby site). Propagules that failed to establish with root growth 1 week after planting were replaced by another propagule of the same cohort. Some maternal trees yielded more propagules than others, so genotypes were assigned to plots in a stratified random design taking number of available propagules per genotype into account. This resulted in uneven representation of the different genotypes. The number of replicate monoculture plots per genotype ranged from 4 to 6. In the three genotype treatment, each genotype was represented in 9–17 plots. In the six genotype treatment, each genotype was represented in 6–13 plots. There were 81 plots in total: 39 plots were planted as monocultures (12 propagules from one genotype), 29 plots were planted with three genotypes (four propagules from each), and 13 plots were planted with six genotypes (two propagules from each). With a pool of eight genotypes, there are more possible combinations of three genotypes compared to six genotypes, hence why more three genotype plots were planted. In June 2016, 1 month prior to our soil sampling, mean tree height within experimental plots was 1.9 m and 88% of trees initially planted had survived. All trees within experimental plots had produced multiple prop roots and most trees had reached reproductive maturity.

### 2.3 Soil sampling

In August 2016, almost five years after the experiment was set up, we sampled soil from 47 of the original experimental plots (Figure 1). The samples were collected in the summer as this is when we would expect the bacterial community to be most active at this site due to warm temperatures and increased exposure to oxygen (compared to late autumn/winter when the soil surface is not exposed at low tide). To test for genotype effects on soil bacterial communities, we sampled 24 of the monoculture plots (three replicates of each genotype). To test for genotypic diversity effects, we also sampled 12 plots that had been planted with three genotypes, and 11 plots that had been planted with six genotypes. We only sampled from plots whose identity could be confirmed by above ground tags in the field. Some tags had become buried in the time since planting and excavation of the below ground tags would have caused significant disturbance to the soil and associated microbial communities. Six subsample soil cores were collected in each plot from random locations among the *R. mangle* prop roots, to a depth of approximately 10 cm, using corers made from sterile 50 ml syringes with the end cut off. The cores for each plot were placed in a single ziplock bag and mixed to form a composite sample of bulk soil. Five control soil samples (also consisting of six subsamples) were collected from mudflat areas across the site that were at least 2 m from the nearest visible tree prop root or pneumatophore. Soil layers below 10 cm were not sampled as oxygen concentrations and hence, microbial activity, rapidly decline with depth from the surface in mangrove soils (Booth et al., 2019). Pore water samples were extracted under suction from each plot (and control location) using a sipper (McKee et al., 1988) and collected into 25 ml scintiallation vials. The GPS coordinates and number of remaining trees were recorded for each sampled plot. All samples were collected during morning low tide and were kept on ice in a cool box in the field and during transportation to the laboratory.

### 2.4 Soil and bacterial analyses

Pore water pH and salinity were measured on the day of collection, after the pore water had equilibrated to room temperature, using an ExStik pH meter (Extech) and an ORA 1SB analog refractometer (Kern Optics) respectively. A subsample from each composite soil sample was oven‐dried at 60°C for 48 hr then ground in a Mixer Mill MM400 (Retsch GmbH). Total % carbon and nitrogen were measured by dry combustion of 10 mg of the ground soil using a Vario EL Cube (Elementar Analysensysteme GmbH). To assess whether there were gradients of pH, salinity, carbon, or nitrogen at the site, which may have affected the outcome of the study, we mapped each of these points in ArcGIS 10.4.1 (ESRI) and used inverse distance weighting in the Spatial Analyst toolkit to interpolate concentrations across the experimental site.

Soil DNA extractions were performed on the day of sample collection using the PowerLyzer PowerSoil DNA Isolation Kit (MoBio Laboratories Inc.) following the manufacturer's protocol except that we used twice the amount of recommended fresh soil (0.5 g) for each extraction. DNA quality and yield were checked using an Epoch microplate spectrophotometer (BioTek Instruments).

The T‐RFLP community fingerprinting method was used to compare soil bacterial community composition and taxa richness between the plots. While T‐RFLP cannot provide taxonomic identification of the bacterial community, it is an established molecular tool which can yield results at a level of resolution comparable to metabarcode sequencing with Illumina Miseq when studying community shifts in plant‐associated microbiomes (Johnston‐Monje & Lopez Mejia, 2020). The 16S rRNA gene was amplified in the soil DNA samples using primers 63F (5′‐CAGGCCTAACACATGCAAGTC‐3) labelled with 6‐FAM fluorescent dye at the 5′end and 530R (5′‐GTATTACCGCGGCTGCTG‐3′) as per Thomson et al. (2010). Each 25 µl reaction contained molecular biology grade water, Standard Taq Reaction Buffer (10 mM Tris‐HCl, 1.5 mM MgCl_2_, 50 mM KCl, pH 8.3 (New England BioLabs)), 0.2 µM of each primer, 1x Bovine Serum Albumin (Promega), 200 µM of deoxynucleoside triphosphates (Roche) and 0.875 U Taq polymerase (New England BioLabs) to which 20–50 ng of template DNA was added. Negative PCR controls were also prepared in the same way except instead of adding DNA from the soil samples, 1 µl of sterile water was added. PCR amplification was carried out in a SimpliAmp thermal cycler (Thermo Fisher Scientific) under the following conditions: 94°C for 1 min 30 s; followed by 30 cycles of 94°C for 45 s, 55°C for 1 min and 72°C for 3 min; followed by 10 min extension at 72°C. PCR products were purified using ethanol precipitation and resuspended in 15 µl molecular biology grade water. 5 µl purified PCR product was digested for 2 hr at 37°C using the MspI enzyme (Promega). Samples were purified again using ethanol precipitation and resuspended in 10 µl molecular biology grade water. 1 µl of the final sample was then mixed with 8.6 µl Hi‐Di formamide and 0.4 µl GeneScan 600 LIZ size standard for fragment sizing using an ABI 3730 (Applied Biosystems).

The resulting data were analyzed using GeneMapper version 4.1 (Applied Biosystems) to score peaks over 150 fluorescence units in the range 50–500 bp and assign bins. This process resulted in a binary matrix file with presence/absence of each bin within each sample. The peak profile of each sample was visually checked and manually edited where peaks were observed that were not detected by the software. All DNA samples were run in duplicate PCRs and only bins that appeared in both replicates were included in the final binary matrix. Only bins that occurred in two or more samples were included in analyses.

### 2.5 Statistical analyses

All statistical analyses were performed in R v3.5.2 (R Core Team, 2018) using the vegan package (Oksanen et al., 2016) unless otherwise stated. All figures were made using the ggplot2 (Wickham, 2016) and ggpubr (Kassambara, 2018) packages.

To compare soil bacterial community profiles, Jaccard similarity matrices were constructed and visualized with nonmetric multidimensional scaling (NMDS) using the metaMDS function. The adonis function was used to perform permutational multivariate analysis of variance (PERMANOVA) with 1,000 permutations to test for significant effects of genotypic identity or genetic diversity on bacterial community composition. As terms are added sequentially, all measured environmental variables (total soil nitrogen and carbon, porewater pH and salinity, and number of trees remaining in the plot) were included in the model before the treatment so that variability attributed to the environmental variables was removed before testing for treatment (genotypic identity or genetic diversity) effects. Environmental vectors were fitted to the NMDS using the envfit function. When testing for genotypic identity, only monoculture plots were included in the analysis and genotypic identity was nested within origin site of the propagules. Mudflat soil samples were not included in the PERMANOVAs testing for genotypic identity or genetic diversity effects. Homogeneity of group dispersion (variance) was measured using the betadisper function and differences between groups were tested using the permutest.betadisper function.

The number of T‐RFLP bins per sample was used as a proxy for bacterial taxa richness. Kruskal–Wallis tests were used to test whether bacterial taxa richness or any of the measured environmental variables differed between the treatments. When significant differences were found, post hoc Dunn tests were performed using the dunnTest function in the FSA package (Ogle et al., 2020) with p‐values adjusted for multiple testing using the Benjamini–Hochberg method (Benjamini & Hochberg, 1995).

## 3 RESULTS

Due to the hydrological flow at the site, gradients of pore water salinity, total soil nitrogen, and total soil carbon were observed across plots in the interpolation maps (Figure S1). However, the random distribution of the experimental plots meant that these variables did not significantly differ among the diversity treatments or among the genotypes in the monoculture plots when tested with Kruskal–Wallis tests (Table 1); as such, these gradients should not confound our results. Within the treatment plots, pore water salinity ranged from 35‰ to 55‰, total soil carbon ranged from 3.97% to 8.84%, and total soil nitrogen ranged from 0.07% to 0.49%. There was no obvious pattern of pore water pH across the site, which ranged from 6.70 to 7.59.

**TABLE 1:** Results of Kruskal–Wallis tests for differences in soil bacterial taxa richness or measured environmental variables between *Rhizophora mangle* genetic diversity treatments (1, 3, and 6 maternal genotypes), and between maternal genotypes (8 total) in monoculture plots

	Monoculture plots			All treatment plots		
	*χ* ^2^	*df*	*p*	*χ* ^2^	*df*	*p*
Taxa richness	3.524	7	0.833	3.135	2	0.209
Pore water pH	13.047	7	0.071	0.375	2	0.829
Pore water salinity	12.244	7	0.093	0.196	2	0.907
Number of trees	5.036	7	0.656	1.485	2	0.476
Total soil carbon	1.680	7	0.975	5.550	2	0.062
Total soil nitrogen	1.875	7	0.966	2.345	2	0.310

An average of 10 trees remained across the sampled treatment plots (range 5–12 trees) and there was no difference in propagule survival between the treatments at this stage of the experiment (Table 1). Due to the amount of plant growth since the experiment had been setup and the difficulty of locating ID tags, we were unable to identify which of the original propagules were no longer present. Thus we could not confirm the diversity level remaining in all plots at the time of sampling. None of the environmental variables measured significantly differed between the mudflat and the treatment plots.

### 3.1 Genotypic identity

**FIGURE 2:**
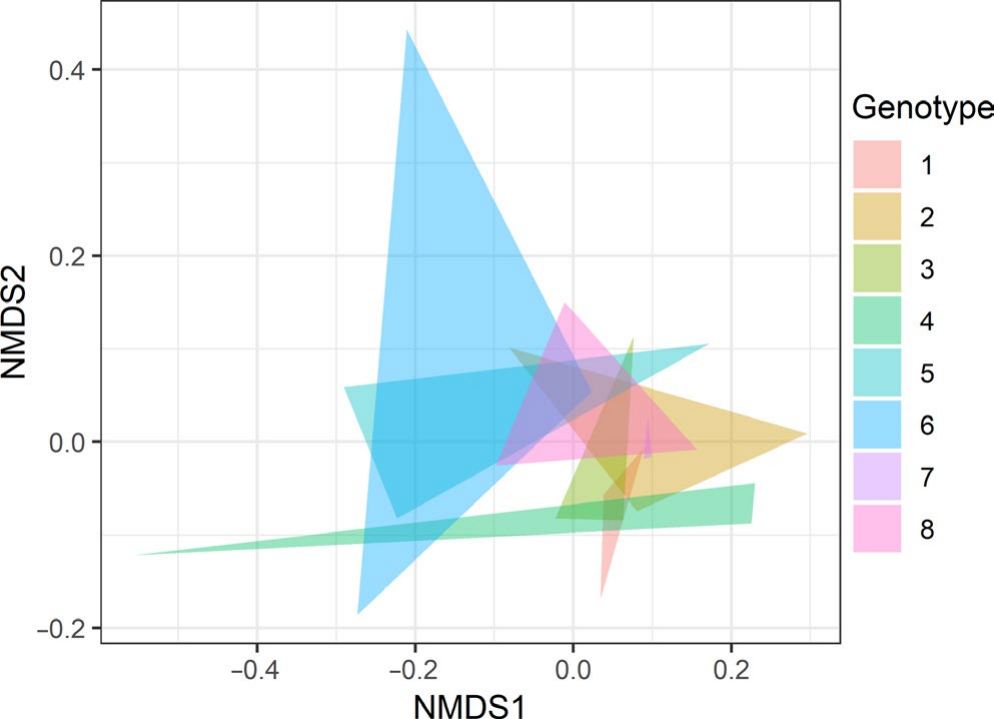
NMDS ordination plot based on the Jaccard's similarity index of bacterial communities examined by 16S T‐RFLP in soil samples collected within monoculture plots (stress = 0.121). Each color represents a different *Rhizophora mangle* maternal genotype and each triangle corner is one plot of that genotype

Within the monoculture plots, PERMANOVA detected a significant relationship between the maternal genotypic identity of *R. mangle* (when nested within origin site) and the associated soil bacterial community (Table 2), with genotypic identity explaining 34% of the variation in bacterial community composition. This was independent of origin site of the propagules, which was not found to be a significant factor explaining bacterial community composition. The ordination plot (Figure 2) reveals that while some of the genotypes cluster quite closely together, indicating that they have similar soil bacteria communities, a few genotypes occupied space that was distinct from some of the other genotypes. Bacterial community composition within the monoculture plots was also significantly related to total soil nitrogen (Table 2). Bacterial taxa richness and the measured environmental variables did not differ among genotypes (Table 1), and there was no detectable difference in group dispersion of soil bacterial communities between genotypes (*F*_7,16_ = 0.957, *p* = 0.535).

**TABLE 2:** Permutational multivariate analysis of variance (PERMANOVA) test results for effects of *Rhizophora mangle* maternal genotypic identity, maternal origin site, and environmental variables within monoculture plots on the Jaccard similarity index of soil bacterial communities based on 16S T‐RFLP profiling

	*df*	Sums of squares	Mean squares	Pseudo *F*	*R* ^2^	*p*
Soil total N %	**1**	**0.073**	**0.073**	**2.560**	**0.090**	**0.019**
Soil total C %	1	0.012	0.012	0.433	0.015	0.913
Pore water pH	1	0.035	0.035	1.212	0.042	0.269
Pore water salinity	1	0.032	0.032	1.129	0.040	0.316
Number of trees	1	0.040	0.040	1.406	0.049	0.207
Origin site	1	0.028	0.028	0.978	0.034	0.618
Origin: Genotypes	**6**	**0.282**	**0.047**	**1.641**	**0.345**	**0.031**
Residuals	11	0.315	0.029		0.385	
Total	23	0.818			1.000	

Note

Results that are significant at the *p* < 0.05 level are indicated in bold.

### 3.2 Genetic diversity

**FIGURE 3:**
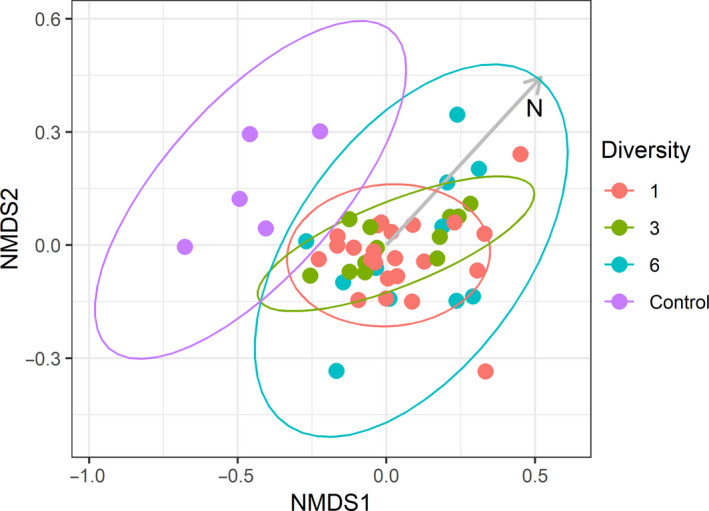
NMDS ordination plot based on the Jaccard's similarity index of bacterial communities examined by 16S T‐RFLP in soil samples collected from plots of *Rhizophora mangle* with different levels of maternal genotypic diversity and control samples collected from the surrounding mudflats (stress = 0.137). The only fitted environmental vector found to be significantly related to soil microbial community composition (total soil N) is shown with the gray arrow

Tree presence had a clear effect on soil bacterial community composition with mudflat samples clustering apart from the samples collected within the treatment plots (Figure 3). However, within the experimental plots, there was no significant difference among the *R. mangle* genetic diversity treatments (Table 3). Of the environmental variables measured, only total soil nitrogen was significantly related to bacterial community composition (Table 3). Although the six genotype treatment appeared to be more variable, there was no significant difference in group dispersion between the genetic diversity treatments (*F*_2,44_ = 2.065, *p* = 0.169; Figure 2).

**TABLE 3:** Permutational multivariate analysis of variance (PERMANOVA) test results for effects of *Rhizophora mangle* genetic diversity (1, 3, or 6 maternal genotypes) and environmental variables on Jaccard's similarity index of soil bacterial communities based on 16S T‐RFLP profiling

	*df*	Sums of squares	Mean squares	Pseudo *F*	*R* ^2^	*p*
Soil total N %	**1**	**0.266**	**0.266**	**7.872**	**0.147**	**0.001**
Soil total C %	1	0.032	0.032	0.935	0.017	0.444
Pore water pH	1	0.031	0.031	0.912	0.017	0.476
Pore water salinity	1	0.033	0.033	0.984	0.018	0.411
Number of trees	1	0.051	0.051	1.521	0.028	0.144
Diversity treatment	2	0.081	0.040	1.192	0.044	0.261
Residuals	39	1.319	0.034		0.728	
Total	46	1.813			1.000	

Note

Results that are significant at the *p* < 0.05 level are indicated in bold.

**FIGURE 4:**
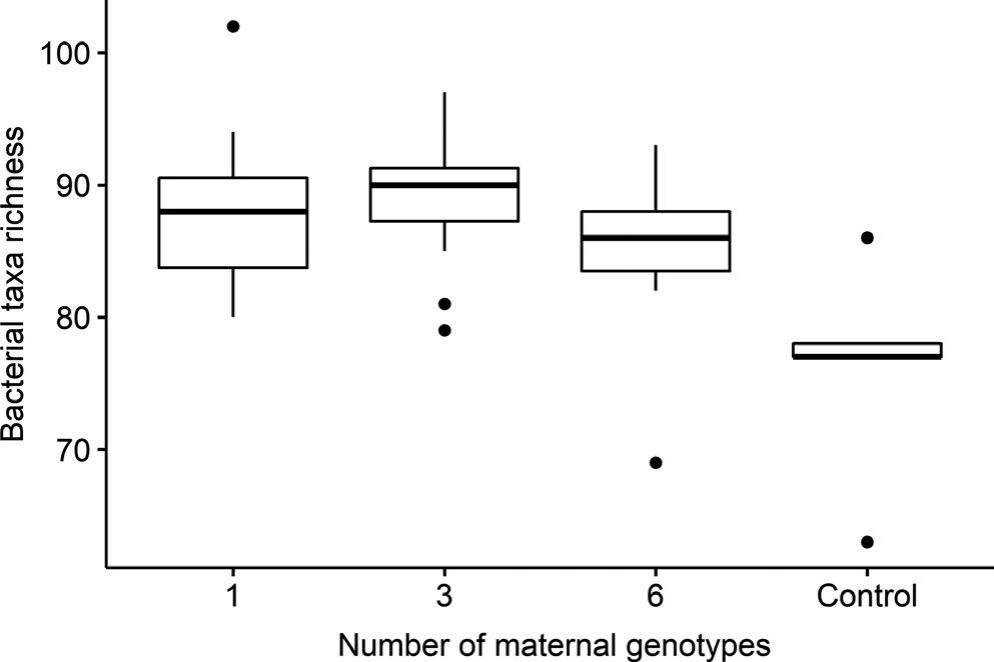
Box plot depicting bacterial taxa richness of bacterial communities, as indicated by 16S T‐RFLP profiling, in soil samples collected from plots of *Rhizophora mangle* with different levels of maternal genotypic diversity and control samples collected from surrounding mudflat areas. The boxes show the median between the first and third quartiles and the whiskers extend up to 1.5 x the interquartile range.

Bacterial taxa richness trended toward a humped distribution, increasing from monocultures to the three genotype treatment, then falling back in the six genotype treatment (Figure 4), but this relationship was not significant (Table 1). The measured environmental variables did not differ between genetic diversity treatments (Table 1). When the mudflat samples were included in the analysis, there was an effect of treatment group (*χ*_2_ = 11.66, *df* = 3, *p* = 0.009) with taxa richness being lower in the mudflat samples compared to the one (*p* = 0.016) and (*p* = 0.005) three genotype treatments.

## 4 DISCUSSION

Our study set out to determine whether genotypic identity and genetic diversity of the neotropical red mangrove, *R. mangle,* influence associated soil bacterial communities. We demonstrate that maternal genotypic identity can drive changes in associated soil bacterial community composition in wetland environments. If different genotypes host different soil bacterial communities, it is plausible that plant community assemblages consisting of more genotypes (i.e., that are more genetically diverse) would harbor more diverse bacterial communities. However, this was not observed in our study, in that richness of the bacterial communities did not differ between the *R. mangle* genetic diversity treatments.

Our findings support those of a growing number of studies showing that plant genotype affects soil microbial community composition (e.g., Aira et al., 2010; Gallart et al., 2018; Schweitzer et al., 2008; Shenton et al., 2016). This relationship has previously been observed in the saltmarsh plant *Spartina alterniflora*, where bacterial rhizosphere communities differed between genotypes and growth form (Zogg et al., 2018), as well as among S. *alterniflora* plants from different populations grown in a common garden (Nie et al., 2010). The results from our study show that even in environments where tidal waters could promote mixing of microbial communities across a site, tree genotype still exerts a detectable influence on the composition of bacterial communities in bulk soils.

In wetlands, the rhizosphere could be particularly important in controlling bacterial community diversity and composition. Higher bacterial diversity observed in *S. alterniflora* rhizosphere compared to bulk soil has been attributed to the plant roots providing oxygen and labile organic compounds in an environment lacking both (Zogg et al., 2018). Our finding that bacterial taxa richness was higher in experimental plots compared to the surrounding mudflats lends support to this suggestion. The root morphology of *R. mangle* is highly adapted for survival in anoxic soil environments and their prop roots can transport oxygen belowground to the rest of the root system (Scholander et al., 1955). In such a plant, any differences in root morphology among genotypes could be expected to have a substantial influence on the composition of associated soil bacterial communities by controlling the supply of oxygen and exudates to the rhizosphere. Variations among genotypes in the chemical composition of exudates (Micallef et al., 2009), or the quantity and quality of leaf litter (Schweitzer et al., 2005), could also drive changes in the bacterial community.

In contrast to Schweitzer et al.'s (2011) study, we did not find an effect of plant genetic diversity on associated bacterial community composition. Schweitzer et al. (2011) found that natural stand genetic diversity was linked to microbial community composition as well as measures related to microbial soil functioning including exoenzyme activity and inorganic nitrogen concentrations. Nitrogen cycling has also been shown to be affected by plant genetic diversity in a pot experiment through increased root litter nitrogen content and decomposition rates compared with litter produced by sibling groups (Semchenko et al., 2017). Although our study and Schweitzer et al.'s (2011) did not find any relationships between plant genetic diversity and total soil nitrogen content, Schweitzer et al. (2011) detected unimodal relationships between genetic diversity and soil nitrate and ammonium concentrations (which we did not measure). While the trees in the experiment plots had reached maturity by the time of our soil sampling, it is possible that a longer time scale is needed before small effects on nutrient cycling become detectable. We suggest that further investigations are warranted to evaluate the relationships between plant genetic diversity, soil microbial community composition, and processes of nitrogen cycling across different biomes in field conditions.

We did not observe any differences in bacterial taxa richness between *R. mangle* genotypes or genetic diversity treatments. While a more diverse plant community might be expected to support a more diverse soil bacterial community, evidence for this is lacking, suggesting that other environmental factors may have a stronger role in regulating soil bacterial diversity (Fierer & Jackson, 2006; Prober et al., 2015; Wardle, 2006). However, the presence of *R. mangle* was found to have some influence on bacterial taxa richness, with significantly higher richness found in the treatment plots compared to the surrounding mudflats. Our bacterial community profiles were based on presence/absence data, so we were only able to assess richness of T‐RFLP fragments as a measure of diversity. Next generation sequencing could be used to determine abundance and evenness of different bacterial taxa in order to calculate diversity indices, which could provide a better understanding of potential effects of plant genetic diversity on bacterial community diversity. Measures of soil function, such as rates of respiration, mineralization, and decomposition, or use of qPCR or metagenomics to assess abundance changes in functional genes could also be used to ascertain whether nutrient cycling processes are impacted as a result of changes in bacterial community composition.

Our experiment comprised maternal cohorts as a proxy for genotype, and we did not conduct genotype analyses on the propagules or the maternal trees. As such, it is likely that there is a degree of variation within each maternal cohort in addition to variation among cohorts. However, due to self‐fertilization rates of *R. mangle* (Kennedy et al., 2017) in this region, we would expect variation to be greater among maternal cohorts than within. As we do not know how related each of our maternal cohorts are, some combinations of genotypes within the three genotype and six genotype diversity treatments may be less diverse than others. This may have impacted our analyses on effects of genotypic diversity on the soil bacterial community composition. Nevertheless, and despite the limitations of our methodology and frequent mixing of water across the site due to tidal influences, we were still able to detect differences in bacterial communities of bulk soil between *R. mangle* genotypes, which points to the strong effect plants can have on the composition of associated bacterial communities.

As the importance of mangroves is becoming more widely recognized, there has been an increase in mangrove restoration efforts in many regions (Bosire et al., 2008). However, genetic diversity of restored sites can be lower than nearby remnant populations (Granado et al., 2018), even three decades after a localized extinction event (Arnaud‐Haond et al., 2009). Where sites have been replanted with propagules, little consideration has usually been given to obtaining a genetically diverse mixture of propagules to ensure a genetically viable population. The results of our study show that *R. mangle* genotype choice can influence the composition of the associated soil bacterial community, which will likely have wider impacts given the importance of bacteria for the functioning of mangrove ecosystems. The origins of the maternal genotypes used in this study were not very spatially distant and yet we were still able to detect differences in associated bacterial communities among genotypes. We would expect to see similar effects in reforestation projects when propagules are collected across a comparable or larger spatial scale. In the absence of knowing which genotypes may be best in particular environments, we suggest that best practice for replanting efforts should aim to increase the genetic diversity of their planting stock to improve chances of successfully establishing a diverse mangrove community. As loss of plant genetic variation associated with key phenotypic traits can exacerbate the impact of environmental change on ecosystem functions provided by soil microbes (Hines et al., 2014), further work is needed to understand the potential implications of genetic diversity loss on important soil functions associated with carbon and nutrient cycling. Changes to the rate of decomposition of organic matter in these environments could affect not only their carbon storage capacity, but also their ability to keep up with sea level rise through vertical accretion of the soil.

## CONFLICT OF INTEREST

None declared.

## AUTHOR CONTRIBUTIONS

**Hayley Craig:** Conceptualization (equal); formal analysis (lead); investigation (lead); methodology (equal); writing – original draft (lead). **John Paul Kennedy:** Investigation (supporting); writing – review and editing (supporting). **Donna J. Devlin:** Conceptualization (equal); methodology (equal); supervision (supporting); writing – review and editing (supporting). **Richard D. Bardgett:** Supervision (equal); writing – review and editing (supporting). **Jennifer K. Rowntree:** Conceptualization (equal); formal analysis (supporting); supervision (equal); writing – review and editing (supporting).

## Supporting information

Figure S1

## Data Availability

Data associated with this manuscript is available from the Dryad Digital Repository (https://doi.org/10.5061/dryad.5tb2rbp2x).

## References

[ece36989-bib-0001] Aira, M. , Gómez‐Brandón, M. , Lazcano, C. , Bååth, E. , & Domínguez, J. (2010). Plant genotype strongly modifies the structure and growth of maize rhizosphere microbial communities. Soil Biology and Biochemistry, 42(12), 2276–2281. 10.1016/j.soilbio.2010.08.029

[ece36989-bib-0002] Alongi, D. M. (1988). Bacterial productivity and microbial biomass in tropical mangrove sediments. Microbial Ecology, 15(1), 59–79. 10.1007/BF02012952 24202863

[ece36989-bib-0003] Alongi, D. M. (2012). Carbon sequestration in mangrove forests. Carbon Management, 3(3), 313–322. 10.4155/cmt.12.20

[ece36989-bib-0004] Andreote, F. D. , Jiménez, D. J. , Chaves, D. , Dias, A. C. F. , Luvizotto, D. M. , Dini‐Andreote, F. , Fasanella, C. C. , Lopez, M. V. , Baena, S. , Taketani, R. G. , & de Melo, I. S. (2012). The microbiome of Brazilian mangrove sediments as revealed by metagenomics. PLoS One, 7(6), e38600 10.1371/journal.pone.0038600 22737213 PMC3380894

[ece36989-bib-0005] Arnaud‐Haond, S. , Duarte, C. M. , Teixeira, S. , Massa, S. I. , Terrados, J. , Tri, N. H. , Hong, P. N. , & Serrão, E. A. (2009). Genetic recolonization of mangrove: Genetic diversity still increasing in the Mekong delta 30 years after Agent Orange. Marine Ecology Progress Series, 390, 129–135. 10.3354/meps08183

[ece36989-bib-0006] Balvanera, P. , Pfisterer, A. B. , Buchmann, N. , He, J. S. , Nakashizuka, T. , Raffaelli, D. , & Schmid, B. (2006). Quantifying the evidence for biodiversity effects on ecosystem functioning and services. Ecology Letters, 9(10), 1146–1156. 10.1111/j.1461-0248.2006.00963.x 16972878

[ece36989-bib-0007] Bardgett, R. , & van der Putten, W. (2014). Belowground biodiversity and ecosystem functioning. Nature, 515, 505–511. 10.1038/nature13855 25428498

[ece36989-bib-0008] Benjamini, Y. , & Hochberg, Y. (1995). Controlling the false discovery rate: A practical and powerful approach to multiple testing. Journal of the Royal Statistical Society, 57(1), 289–300.

[ece36989-bib-0009] Booth, J. M. , Fusi, M. , Marasco, R. , Mbobo, T. , & Daffonchio, D. (2019). Fiddler crab bioturbation determines consistent changes in bacterial communities across contrasting environmental conditions. Scientific Reports, 9, 3749 10.1038/s41598-019-40315-0 30842580 PMC6403291

[ece36989-bib-0010] Bosire, J. O. , Dahdouh‐Guebas, F. , Walton, M. , Crona, B. I. , Lewis, R. R. , Field, C. , Kairo, J. G. , & Koedam, N. (2008). Functionality of restored mangroves: A review. Aquatic Botany, 89(2), 251–259. 10.1016/j.aquabot.2008.03.010

[ece36989-bib-0011] Boyden, S. , Binkley, D. , & Stape, J. L. (2008). Competition among Eucalyptus trees depends on genetic variation and resource supply. Ecology, 89(10), 2850–2859. 18959322 10.1890/07-1733.1

[ece36989-bib-0012] Bruschi, P. , Angeletti, C. , González, O. , Adele Signorini, M. , & Bagnoli, F. (2014). Genetic and morphological variation of *Rhizophora mangle* (red mangrove) along the northern Pacific coast of Nicaragua. Nordic Journal of Botany, 32(3), 320–329.

[ece36989-bib-0013] Cardinale, B. J. , Matulich, K. L. , Hooper, D. U. , Byrnes, J. E. , Duffy, E. , Gamfeldt, L. , Balvanera, P. , O’Connor, M. I. , & Gonzalez, A. (2011). The functional role of producer diversity in ecosystems. American Journal of Botany, 98(3), 572–592. 10.3732/ajb.1000364 21613148

[ece36989-bib-0014] Cebrian, J. (1999). Patterns in the fate of production in plant communities. The American Naturalist, 154(4), 449–468. 10.1086/303244 10523491

[ece36989-bib-0015] Crutsinger, G. M. , Collins, M. D. , Fordyce, J. A. , Gompert, Z. , Nice, C. C. , & Sanders, N. J. (2006). Plant genotypic diversity predicts community structure and governs an ecosystem process. Science, 313(5789), 966–968. 10.1126/science.1128326 16917062

[ece36989-bib-0016] Crutsinger, G. M. , Souza, L. , & Sanders, N. J. (2008). Intraspecific diversity and dominant genotypes resist plant invasions. Ecology Letters, 11(1), 16–23. 17971172 10.1111/j.1461-0248.2007.01118.x

[ece36989-bib-0017] Devlin, D. J. (2004). Analyses of the relationship between a parasitic beetle (*Coccotrypes rhizophorae*) and a host plant, the red mangrove (*Rhizophora mangle*). PhD Thesis, University of Louisiana at Lafayette.

[ece36989-bib-0018] Donato, D. C. , Kauffman, J. B. , Murdiyarso, D. , Kurnianto, S. , Stidham, M. , & Kanninen, M. (2011). Mangroves among the most carbon‐rich forests in the tropics. Nature Geoscience, 4, 293–297. 10.1038/ngeo1123

[ece36989-bib-0019] Duffy, J. , Godwin, C. , & Cardinale, B. (2017). Biodiversity effects in the wild are common and as strong as key drivers of productivity. Nature, 549, 261–264. 10.1038/nature23886 28869964

[ece36989-bib-0020] Farnsworth, E. J. (1998). Issues of spatial, taxonomic and temporal scale in delineating links between mangrove diversity and ecosystem function. Global Ecology and Biogeography Letters, 7(1), 15–25. 10.2307/2997694

[ece36989-bib-0021] Fierer, N. , & Jackson, R. B. (2006). The diversity and biogeography of soil bacterial communities. Proceedings of the National Academy of Sciences of the United States of America, 103(3), 626–631. 16407148 10.1073/pnas.0507535103 PMC1334650

[ece36989-bib-0022] Frankham, R. (2005). Genetics and extinction. Biological Conservation, 126, 131–140. 10.1016/j.biocon.2005.05.002

[ece36989-bib-0023] Gallart, M. , Adair, K. L. , Love, J. , Meason, D. F. , Clinton, P. W. , Xue, J. , & Turnbull, M. H. (2018). Genotypic variation in *Pinus radiata* responses to nitrogen source are related to changes in the root microbiome. FEMS Microbiology Ecology, 94(6), fiy071 10.1093/femsec/fiy071 29688427

[ece36989-bib-0024] Granado, R. , Neta, L. C. P. , Nunes‐Freitas, A. F. , Voloch, C. M. , & Lira, C. F. (2018). Assessing genetic diversity after mangrove restoration in Brazil: Why is it so important? Diversity, 10, 27 10.3390/d10020027

[ece36989-bib-0025] Gratani, L. (2014). Plant phenotypic plasticity in response to environmental factors. Advances in Botany, 2014, 208747 10.1155/2014/208747

[ece36989-bib-0026] Helm, A. , Oja, T. , Saar, L. , Takkis, K. , Talve, T. , & Pärtel, M. (2009). Human influence lowers plant genetic diversity in communities with extinction debt. Journal of Ecology, 97(6), 1329–1336. 10.1111/j.1365-2745.2009.01572.x

[ece36989-bib-0027] Hines, J. , Reyes, M. , Mozder, T. J. , & Gessner, M. O. (2014). Genotypic trait variation modifies effects of climate warming and nitrogen deposition on litter mass loss and microbial respiration. Global Change Biology, 20(12), 3780–3789. 10.1111/gcb.12704 25099691

[ece36989-bib-0028] Holguin, G. , Vazquez, P. , & Bashan, Y. (2001). The role of sediment microorganisms in the productivity, conservation, and rehabilitation of mangrove ecosystems: An overview. Biology and Fertility of Soils, 33(4), 265–278. 10.1007/s003740000319

[ece36989-bib-0029] Hooper, D. U. , Adair, E. C. , Cardinale, B. J. , Byrnes, J. E. K. , Hungate, B. A. , Matulich, K. L. , Gonzalez, A. , Duffy, J. E. , Gamfeldt, L. , & Connor, M. I. (2012). A global synthesis reveals biodiversity loss as a major driver of ecosystem change. Nature, 486(7401), 105–108. 10.1038/nature11118 22678289

[ece36989-bib-0030] Hooper, D. U. , Chapin, F. S. , Ewel, J. J. , Hector, A. , Inchausti, P. , Lavorel, S. , Lawton, J. H. , Lodge, D. M. , Loreau, M. , Naeem, S. , Schmid, B. , Setälä, H. , Symstad, A. J. , Vandermeer, J. , & Wardle, D. A. (2005). Effects of biodiversity on ecosystem functioning: A consensus of current knowledge. Ecological Monographs, 75(1), 3–35. 10.1890/04-0922

[ece36989-bib-0031] Hughes, A. R. , Inouye, B. D. , Johnson, M. T. J. , Underwood, N. , & Vellend, M. (2008). Ecological consequences of genetic diversity. Ecology Letters, 11(6), 609–623. 10.1111/j.1461-0248.2008.01179.x 18400018

[ece36989-bib-0032] IPBES (2019). Summary for policymakers of the global assessment report on biodiversity and ecosystem services of the Intergovernmental Science‐Policy Platform on Biodiversity and Ecosystem Services In Díaz S., Settele J., Brondízio E. S., Ngo H. T., Guèze M., Agard J., Arneth A., Balvanera P., Brauman K. A., Butchart S. H. M., Chan K. M. A., Garibaldi L. A., Ichii K., Liu J., Subramanian S. M., Midgley G. F., Miloslavich P., Molnár Z., Obura D., … Zayas C. N. , (3–5). IPBES Secretariat https://ipbes.net/sites/default/files/downloads/spm_unedited_advance_for_posting_htn.pdf.

[ece36989-bib-0033] Johnston‐Monje, D. , & Lopez Mejia, J. (2020). Botanical microbiomes on the cheap: Inexpensive molecular fingerprinting methods to study plant‐associated communities of bacteria and fungi. Applications in Plant Sciences, 8(4), e11334. 32351795 10.1002/aps3.11334 PMC7186905

[ece36989-bib-0034] Jump, A. S. , Marchant, R. , & Peñuelas, J. (2009). Environmental change and the option value of genetic diversity. Trends in Plant Science, 14(1), 51–58. 10.1016/j.tplants.2008.10.002 19042147

[ece36989-bib-0035] Kassambara, A. (2018). ggpubr: “ggplot2” based publication ready plots. R package version 0.2. Retrieved from https://CRAN.R‐project.org/package=ggpubr

[ece36989-bib-0036] Kennedy, J. P. , Garavelli, L. , Truelove, N. K. , Devlin, D. J. , Box, S. J. , Chérubin, L. M. , & Feller, I. C. (2017). Contrasting genetic effects of red mangrove (*Rhizophora mangle* L.) range expansion along West and East Florida. Journal of Biogeography, 44(2), 335–347.

[ece36989-bib-0037] Koricheva, J. , & Hayes, D. (2018). The relative importance of plant intraspecific diversity in structuring arthropod communities: A meta‐analysis. Functional Ecology, 32(7), 1704–1717. 10.1111/1365-2435.13062

[ece36989-bib-0038] Liu, L. , Zhu, K. , Wurzburger, N. , & Zhang, J. (2020). Relationships between plant diversity and soil microbial diversity vary across taxonomic groups and spatial scales. Ecosphere, 11(1), e02999 10.1002/ecs2.2999

[ece36989-bib-0039] Loreau, M. , Naeem, S. , Inchausti, P. , Bengtsson, J. , Grime, J. P. , Hector, A. , Hooper, D. U. , Huston, M. A. , Raffaelli, D. , Schmid, B. , Tilman, D. , & Wardle, D. A. (2001). Biodiversity and ecosystem functioning: Current knowledge and future challenges. Science, 294(5543), 804–808. 10.1126/science.1064088 11679658

[ece36989-bib-0040] Lowenfeld, R. , & Klekowski, E. J. (1992). Mangrove Genetics. I. Mating system and mutation rates of *Rhizophora mangle* in Florida and San Salvador Island, Bahamas. International Journal of Plant Sciences, 153(3), 394–399. 10.1086/297043

[ece36989-bib-0041] Maron, P. A. , Sarr, A. , Kaisermann, A. , Lévêque, J. , Mathieu, O. , Guigue, J. , Karimi, B. , Bernard, L. , Dequiedt, S. , Terrat, S. , Chabbi, A. , & Ranjard, L. (2018). High microbial diversity promotes soil ecosystem functioning. Applied and Environmental Microbiology, 84(9), e02738–e2817. 10.1128/AEM.02738-17 29453268 PMC5930326

[ece36989-bib-0042] McGranahan, G. , Balk, D. , & Anderson, B. (2007). The rising tide: Assessing the risks of climate change and human settlements in low elevation coastal zones. Environment and Urbanization, 19(1), 17–37. 10.1177/0956247807076960

[ece36989-bib-0043] McKee, K. L. (1993). Soil physicochemical patterns and mangrove species distribution‐reciprocal effects? Journal of Ecology, 81(3), 477–487. 10.2307/2261526

[ece36989-bib-0044] McKee, K. L. , Mendelssohn, I. A. , & Hester, M. W. (1988). Reexamination of pore water sulfide concentrations and redox potentials near the aerial roots of *Rhizophora mangle* and *Avicennia germinans* . American Journal of Botany, 75(9), 1352–1359. 10.1002/j.1537-2197.1988.tb14196.x

[ece36989-bib-0045] Micallef, S. A. , Shiaris, M. P. , & Colon‐Carmona, A. (2009). Influence of *Arabidopsis thaliana* accessions on rhizobacterial communities and natural variation in root exudates. Journal of Experimental Botany, 60(6), 1729–1742. 10.1093/jxb/erp053 19342429 PMC2671628

[ece36989-bib-0046] Newman, D. , & Pilson, D. (2006). Increased probability of extinction due to decreased genetic effective population size: Experimental populations of *Clarkia pulchella* . Evolution, 51(2), 354. 10.1111/j.1558-5646.1997.tb02422.x 28565367

[ece36989-bib-0047] Nie, M. , Gao, L. X. , Yan, J. H. , Fu, X. H. , Xiao, M. , Yang, J. , & Li, B. (2010). Population variation of invasive *Spartina alterniflora* can differentiate bacterial diversity in its rhizosphere. Plant Ecology, 209(2), 219–226.

[ece36989-bib-0048] Ogle, D. H. , Wheeler, P. , & Dinno, A. (2020). FSA: Fisheries Stock Analysis. R package version 0.8.30.9000. Retrieved from https://github.com/droglenc/FSA

[ece36989-bib-0049] Oksanen, J. , Guillaume Blanchet, F. , Kindt, R. , Legendre, P. , Minchin, P. R. , O'Hara, R. B. , Simpson, G. L. , Solymos, P. , Stevens, H. H. , & Wagner, H. (2016). vegan: Community ecology package v 2.4‐0. https://cran.r‐project.org/web/packages/vegan/index.html.

[ece36989-bib-0050] Prieto, I. , Violle, C. , Barre, P. , Durand, J.‐L. , Ghesquiere, M. , & Litrico, I. (2015). Complementary effects of species and genetic diversity on productivity and stability of sown grasslands. Nature Plants, 1(4), 15033 10.1038/nplants.2015.33 27247033

[ece36989-bib-0051] Prober, S. M. , Leff, J. W. , Bates, S. T. , Borer, E. T. , Firn, J. , Harpole, W. S. , Lind, E. M. , Seabloom, E. W. , Adler, P. B. , Bakker, J. D. , Cleland, E. E. , DeCrappeo, N. M. , DeLorenze, E. , Hagenah, N. , Hautier, Y. , Hofmockel, K. S. , Kirkman, K. P. , Knops, J. M. , La Pierre, K. J. , … Fierer, N. (2015). Plant diversity predicts beta but not alpha diversity of soil microbes across grasslands worldwide. Ecology Letters, 18(1), 85–95. 10.1111/ele.12381 25430889

[ece36989-bib-0052] Proffitt, C. E. , Milbrandt, E. C. , & Travis, S. E. (2006). Red mangrove (*Rhizophora mangle*) reproduction and seedling colonization after Hurricane Charley: Comparisons of Charlotte Harbor and Tampa Bay. Estuaries and Coasts, 29(6A), 972–978. 10.1007/BF02798658

[ece36989-bib-0053] Proffitt, C. E. , & Travis, S. E. (2010). Red mangrove seedling survival, growth, and reproduction: Effects of environment and maternal genotype. Estuaries and Coasts, 33(4), 890–901. 10.1007/s12237-010-9265-6

[ece36989-bib-0054] Purahong, W. , Durka, W. , Fischer, M. , Dommert, S. , Schöps, R. , Buscot, F. , & Wubet, T. (2016). Tree species, tree genotypes and tree genotypic diversity levels affect microbe‐mediated soil ecosystem functions in a subtropical forest. Scientific Reports, 6, 36672 10.1038/srep36672 27857198 PMC5114573

[ece36989-bib-0055] R Core Team (2018). R: A language and environment for statistical computing. R Foundation for Statistical Computing.

[ece36989-bib-0056] Raffard, A. , Santoul, F. , Cucherousset, J. , & Blanchet, S. (2019). The community and ecosystem consequences of intraspecific diversity: A meta‐analysis. Biological Reviews, 94(2), 648–661. 10.1111/brv.12472 30294844

[ece36989-bib-0057] Reed, D. H. , & Frankham, R. (2003). Correlation between fitness and genetic diversity. Conservation Biology, 17(1), 230–237. 10.1046/j.1523-1739.2003.01236.x

[ece36989-bib-0058] Reusch, T. B. H. , Ehlers, A. , Hämmerli, A. , & Worm, B. (2005). Ecosystem recovery after climatic extremes enhanced by genotypic diversity. Proceedings of the National Academy of Sciences of the United States of America, 102(8), 2826–2831. 10.1073/pnas.0500008102 15710890 PMC549506

[ece36989-bib-0059] Rowntree, J. K. , & Craig, H. (2019). The contrasting roles of host species diversity and parasite population genetic diversity in the infection dynamics of a keystone parasitic plant. Journal of Ecology, 107(1), 23–33. 10.1111/1365-2745.13050

[ece36989-bib-0060] Salas‐Leiva, D. E. , Mayor‐Durán, V. M. , & Toro‐Perea, N. (2009). Genetic diversity of black mangrove (*Avicennia germinans*) in natural and reforested areas of Salamanca Island Parkway, Colombian Caribbean. Hydrobiologia, 620(1), 17–24.

[ece36989-bib-0061] Scholander, P. F. , van Dam, L. , & Scholander, S. I. (1955). Gas exchange in the roots of mangroves. American Journal of Botany, 42(1), 92–98. 10.1002/j.1537-2197.1955.tb11097.x

[ece36989-bib-0062] Schweitzer, J. , Bailey, J. , Fischer, D. , Leroy, C. , Lonsdorf, E. , Whitham, T. , & Hart, S. (2008). Plant–soil–microorganism interactions: Heritable relationship between plant genotype and associated soil microorganisms. Ecology, 89(3), 773–781. 10.1890/07-0337.1 18459340

[ece36989-bib-0063] Schweitzer, J. A. , Bailey, J. K. , Hart, S. C. , Wimp, G. M. , Chapman, S. K. , & Whitham, T. G. (2005). The interaction of plant genotype and herbivory decelerate leaf litter decomposition and alter nutrient dynamics. Oikos, 110(1), 133–145. 10.1111/j.0030-1299.2005.13650.x

[ece36989-bib-0064] Schweitzer, J. A. , Fischer, D. G. , Rehill, B. J. , Wooley, S. C. , Woolbright, S. A. , Lindroth, R. L. , Whitham, T. G. , Zak, D. R. , & Hart, S. C. (2011). Forest gene diversity is correlated with the composition and function of soil microbial communities. Population Ecology, 53(1), 35–46. 10.1007/s10144-010-0252-3

[ece36989-bib-0065] Semchenko, M. , Saar, S. , & Lepik, A. (2017). Intraspecific genetic diversity modulates plant‐soil feedback and nutrient cycling. New Phytologist, 216(1), 90–98. 10.1111/nph.14653 28608591

[ece36989-bib-0066] Shenton, M. , Iwamoto, C. , Kurata, N. , & Ikeo, K. (2016). Effect of wild and cultivated rice genotypes on rhizosphere bacterial community composition. Rice, 9, 42 10.1186/s12284-016-0111-8 27557607 PMC4996804

[ece36989-bib-0067] Smith, N. (1986). The rise and fall of the estuarine intertidal zone. Estuaries, 9(2), 95–101. 10.2307/1351941

[ece36989-bib-0068] Spalding, M. , Kainuma, M. , & Collins, L. (2010). World atlas of mangroves. Routledge.

[ece36989-bib-0069] Thomson, B. C. , Ostle, N. , McNamara, N. , Bailey, M. J. , Whiteley, A. S. , & Griffiths, R. I. (2010). Vegetation affects the relative abundances of dominant soil bacterial taxa and soil respiration rates in an upland grassland soil. Microbial Ecology, 59(2), 335–343. 10.1007/s00248-009-9575-z 19705192

[ece36989-bib-0070] Verhoeven, K. J. F. , VonHoldt, B. M. , & Sork, V. L. (2016). Epigenetics in ecology and evolution: What we know and what we need to know. Molecular Ecology, 25(8), 1631–1638. 26994410 10.1111/mec.13617

[ece36989-bib-0071] Wagg, C. , Schlaeppi, K. , Banerjee, S. , Kuramae, E. E. , & van der Heijden, M. G. A. (2019). Fungal‐bacterial diversity and microbiome complexity predict ecosystem functioning. Nature Communications, 10, 4841 10.1038/s41467-019-12798-y PMC6813331 31649246

[ece36989-bib-0072] Wardle, D. A. (2006). The influence of biotic interactions on soil biodiversity. Ecology Letters, 9(7), 870–886. 10.1111/j.1461-0248.2006.00931.x 16796577

[ece36989-bib-0073] Wardle, D. A. (2016). Do experiments exploring plant diversity–Ecosystem functioning relationships inform how biodiversity loss impacts natural ecosystems? Journal of Vegetation Science, 27(3), 646–653. 10.1111/jvs.12399

[ece36989-bib-0074] Wickham, H. (2016). ggplot2: Elegant graphics for data analysis. Springer‐Verlag.

[ece36989-bib-0075] Zheng, Q. , Hu, Y. , Zhang, S. , Noll, L. , Böckle, T. , Dietrich, M. , Herbold, C. W. , Eichorst, S. A. , Woebken, D. , Richter, A. , & Wanek, W. (2019). Soil multifunctionality is affected by the soil environment and by microbial community composition and diversity. Soil Biology and Biochemistry, 136, 107521 10.1016/j.soilbio.2019.107521 31700196 PMC6837881

[ece36989-bib-0076] Zhou, Z. , Wang, C. , & Luo, Y. (2020). Meta‐analysis of the impacts of global change factors on soil microbial diversity and functionality. Nature Communications, 11, 3072 10.1038/s41467-020-16881-7 PMC7300008 32555185

[ece36989-bib-0077] Zogg, G. P. , Travis, S. E. , & Brazeau, D. A. (2018). Strong associations between plant genotypes and bacterial communities in a natural salt marsh. Ecology and Evolution, 8(9), 4721–4730. 10.1002/ece3.4105 29760911 PMC5938472

