# Supplementary material for: Effects of maternal genotypic identity and genetic diversity of the red mangrove Rhizophora mangle on associated soil bacterial communities: A field‐based experiment
Source: Ecol Evol. 2020 Nov 10;10(24):13957–67. doi: 10.1002/ece3.6989 (PMC7771162; doi:10.1002/ece3.6989)
Supplement: Supplementary file 1 — Figure S1 [file ECE3-10-13957-s001.pdf]

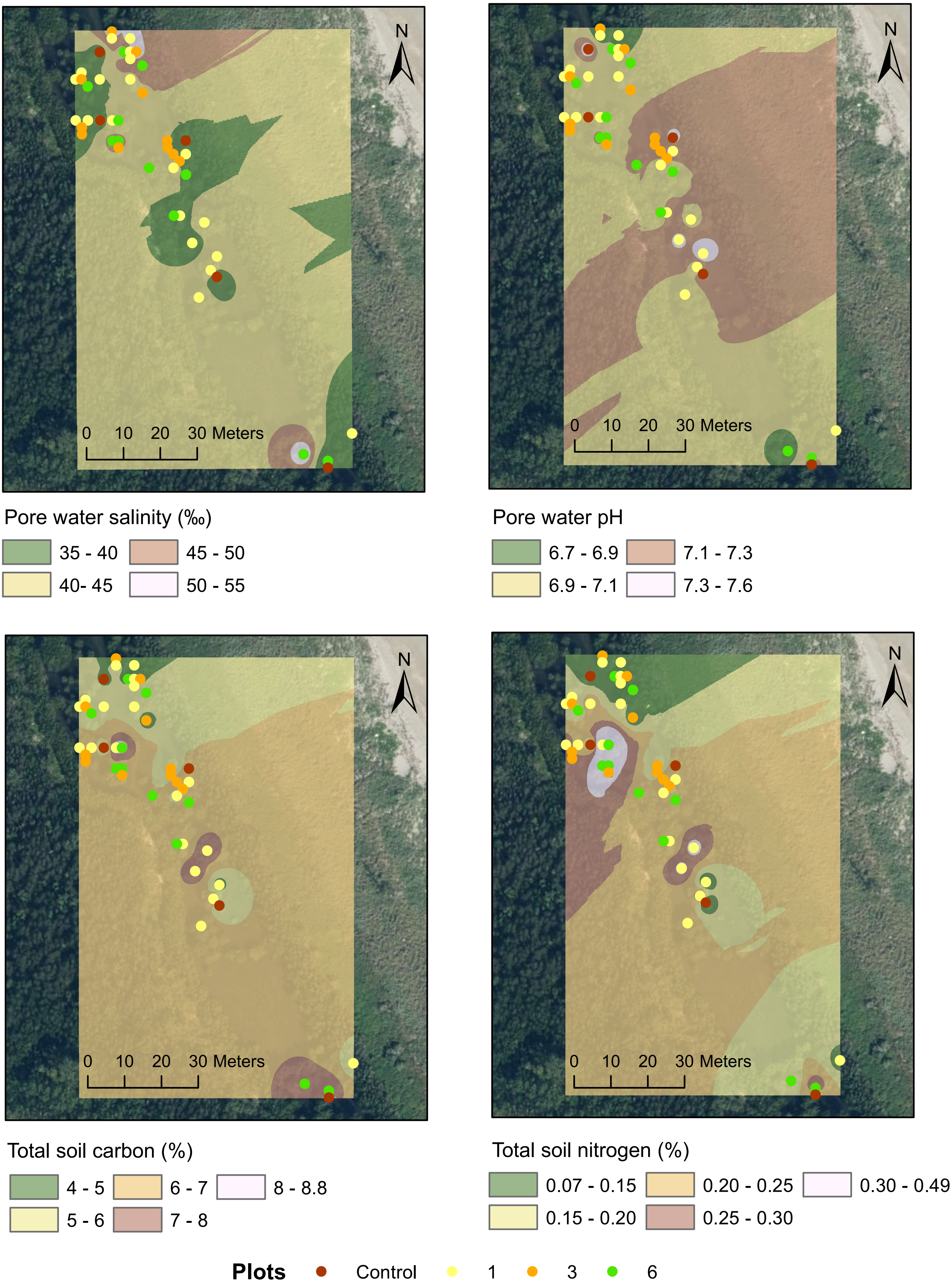

Figure S1. Interpolated soil and porewater variables across the experimental site. Maps produced in ESRI ArcMap 10.4.1. Sampled plot coordinates ± 5 m accuracy.
